# Current trends and applications of virtual reality-assisted music therapy: a scoping review

**DOI:** 10.3389/fpsyg.2026.1814734

**Published:** 2026-07-30

**Authors:** Zhenting Lu, Siyu Long

**Affiliations:** Jiangxi Industry Polytechnic College, Nanchang, Jiangxi, China

**Keywords:** mental health, music therapy, rehabilitation, scoping review, virtual reality

## Abstract

**Introduction:**

With the proliferation of digital technologies, research on virtual reality-assisted music therapy (VR-assisted MT) has expanded across clinical, rehabilitation, and wellness contexts. This scoping review aimed to map the current applications and characteristics of VR-assisted MT, defined as interventions in which music or music therapy is intentionally integrated as a therapeutic component rather than used solely as incidental headset audio.

**Methods:**

The review followed the scoping review framework proposed by Arksey and O’Malley, was further informed by updated JBI guidance, and was reported in accordance with the PRISMA Extension for Scoping Reviews (PRISMA-ScR). Searches were conducted in PubMed, CINAHL Complete, PsycINFO, ScienceDirect, Web of Science, Scopus, ProQuest, China National Knowledge Infrastructure, and Wanfang Data. The search strategy included both abbreviated and unabbreviated terms for virtual reality and music therapy. English- and Chinese-language records published between 1 January 2015 and 30 November 2025 were considered. Formal critical appraisal was not undertaken because the objective was to map the breadth and characteristics of the field; however, study design, intervention reporting, and ethical reporting were considered during eligibility screening and data extraction.

**Results:**

The searches identified 107 records, of which 16 studies were included in the descriptive synthesis. Eleven full-text reports were excluded because they did not report ethics approval or informed consent procedures. The included studies addressed psychological therapy, neurological and motor rehabilitation, pain and symptom management, cognitive rehabilitation, and intervention feasibility or user experience. The available evidence suggested potential benefits for emotional regulation, engagement, pain relief, cognitive function, and motor rehabilitation.

**Discussion:**

VR-assisted MT represents an emerging interdisciplinary field with applications across several therapeutic and rehabilitative contexts. However, the evidence remains preliminary because of small sample sizes, substantial heterogeneity in populations and interventions, incomplete reporting of music therapy protocols, and limited follow-up. Future studies should adopt more rigorous designs and provide clearer reporting of VR characteristics, musical components, therapist involvement, ethical procedures, intervention fidelity, adverse events, and longer-term outcomes.

## 1 Introduction

Music therapy (MT) is a process in which professional music therapists engage individuals or groups in musical activities to address physical, emotional, spiritual, social, and cognitive needs (Johnson et al., 2003). It encompasses a broad spectrum of practices and involves the flexible use of musical elements and techniques to support therapeutic experiences, emotional regulation, social interaction, and cognitive or physical functioning.

MT has shown therapeutic value as a non-pharmacological intervention across different populations and clinical contexts. Previous studies have reported that MT may reduce anxiety in patients with Alzheimer’s disease (Dimitriou et al., 2020), alleviate pain and anxiety in patients undergoing surgery (Gökçek and Kaydu, 2020), enhance social communication skills in children with autism (Sharda et al., 2018), and improve psychotic symptoms, social functioning, and rehabilitation outcomes among long-term hospitalised patients with schizophrenia (Wang et al., 2024).

However, conventional MT still faces several challenges in practical application. Its implementation often relies on the on-site expertise of professionally trained music therapists, which may limit scalability and consistency across service contexts (de Witte et al., 2022). Conventional MT may also face difficulties in achieving stable personalisation and continuous outcome monitoring (Wei and He, 2026). In addition, acoustic conditions, therapeutic settings, intervention formats, and individual responses may influence intervention delivery and outcomes (Rossetti, 2020). Maintaining long-term engagement and immersive therapeutic experience may also be challenging in some contexts (Huang, 2018).

Virtual reality (VR) may offer potential ways to address some of these challenges by providing controllable, repeatable, and interactive therapeutic environments. Huang (2018) defines VR as a technology that generates computer-simulated three-dimensional environments, enabling users to interact with these spaces in real time through dedicated equipment and creating an immersive experience. In the context of music-based intervention, VR can support scenario creation, which refers to the construction of coherent therapeutic contexts, such as natural relaxation landscapes, simulated performance environments, or rehabilitation task spaces, in which music, visual stimuli, and interaction can be aligned with therapeutic goals. Such environments may strengthen presence, support graded exposure, enhance engagement, and enable multisensory feedback during music-based intervention.

VR-assisted MT may therefore be understood as a therapeutic approach in which VR functions as an immersive delivery and interaction environment, while music therapy or a music-based intervention remains the primary therapeutic element. This distinction is important because many VR applications contain background sound, environmental sound effects, or headset audio. Such incidental audio alone was not considered sufficient for inclusion unless music was intentionally designed, selected, or described as part of the therapeutic process or music-based intervention.

Although previous reviews have examined music-based interventions, music for emotion regulation, or reporting quality in music intervention research (Chong et al., 2024; Robb et al., 2018; van Essen et al., 2025), evidence specifically addressing VR-assisted MT remains fragmented across disciplines, populations, intervention formats, and publication types. A scoping review is therefore appropriate for clarifying how this emerging field is currently defined, what intervention models have been tested, which populations and outcomes have been examined, and where methodological and reporting gaps remain.

## 2 Materials and methods

### 2.1 Aims and objectives

This study followed the updated JBI guidance for scoping reviews proposed by Peters et al. (2020) and used a scoping design because the evidence base is emerging, heterogeneous, and distributed across clinical, rehabilitation, music therapy, and human-computer interaction literature. The purpose was not to estimate pooled effectiveness but to map study characteristics, intervention models, target populations, and knowledge gaps. The review addressed four questions: (1) What are the primary application domains and target populations for VR-assisted MT? (2) What research designs have been used? (3) How are VR technologies and music therapy components implemented? (4) What therapeutic processes, outcomes, and reporting gaps are evident?

### 2.2 Design

This scoping review was conducted in accordance with the foundational framework proposed by Arksey and O’Malley (2005), further informed by the methodological guidance for scoping reviews provided by Peters et al. (2020), and reported in accordance with the PRISMA Extension for Scoping Reviews (PRISMA-ScR). A formal review protocol was developed internally before data extraction to specify the research questions, eligibility criteria, databases, search strategy, and extraction variables; however, the protocol was not prospectively registered. This absence of prospective registration is acknowledged as a limitation of the review process.

### 2.3 Search strategy

This scoping review followed PRISMA-ScR reporting guidance. Relevant studies published from 1 January 2015 to 30 November 2025 were retrieved from electronic databases. English-language databases included PubMed, CINAHL Complete, PsycINFO, ScienceDirect, Web of Science, Scopus, and ProQuest. Chinese-language databases comprised China National Knowledge Infrastructure (CNKI) and Wanfang Data. The revised English search string was: (“virtual reality” OR VR OR “VR technology” OR “virtual environment*” OR “immersive virtual reality” OR “mixed reality” OR “extended reality” OR “augmented reality”) AND (“music therapy” OR “music therap*” OR music OR MT OR “music intervention*” OR “music-based intervention*” OR “therapeutic music”) Chinese search terms included 音乐治疗, 音乐疗愈, 音乐干预, 音乐聆听, 治疗性音乐, 虚拟现实, 虚拟现实技术, 虚拟环境, 沉浸式虚拟现实, 混合现实, 增强现实, and 扩展现实. Search queries were adapted to the syntax and indexing functions of each database and were designed to retrieve records from titles, abstracts, and keywords where permitted. The specific search strategy is presented in Table 1.

**Table 1:** Database-specific search strategies.

Database	Search fields	Database-specific search strategy
PubMed	Title / abstract	((“virtual reality” OR VR OR “VR technology” OR “virtual environment*” OR “immersive virtual reality” OR “mixed reality” OR “extended reality” OR “augmented reality”) AND (“music therapy” OR “music therap*” OR music OR MT OR “music intervention*” OR “music-based intervention*” OR “therapeutic music”))
CINAHL Complete	Title / abstract / keywords	((“virtual reality” OR VR OR “VR technology” OR “virtual environment*” OR “immersive virtual reality” OR “mixed reality” OR “extended reality” OR “augmented reality”) AND (“music therapy” OR “music therap*” OR music OR MT OR “music intervention*” OR “music-based intervention*” OR “therapeutic music”))
PsycINFO	Title / abstract	((“virtual reality” OR VR OR “VR technology” OR “virtual environment*” OR “immersive virtual reality” OR “mixed reality” OR “extended reality” OR “augmented reality”) AND (“music therapy” OR “music therap*” OR music OR MT OR “music intervention*” OR “music-based intervention*” OR “therapeutic music”))
ScienceDirect	Title / abstract / keywords	((“virtual reality” OR “immersive virtual reality” OR “mixed reality” OR “extended reality” OR “augmented reality”) AND (“music therapy” OR “music intervention” OR “music-based intervention” OR “therapeutic music”))
Web of Science	Topic	TS = ((“virtual reality” OR VR OR “VR technology” OR “virtual environment*” OR “immersive virtual reality” OR “mixed reality” OR “extended reality” OR “augmented reality”) AND (“music therapy” OR “music therap*” OR music OR MT OR “music intervention*” OR “music-based intervention*” OR “therapeutic music”))
Scopus	Title / abstract / keywords	TITLE-ABS-KEY((“virtual reality” OR VR OR “VR technology” OR “virtual environment*” OR “immersive virtual reality” OR “mixed reality” OR “extended reality” OR “augmented reality”) AND (“music therapy” OR “music therap*” OR music OR MT OR “music intervention*” OR “music-based intervention*” OR “therapeutic music”))
ProQuest	Title / abstract / subject terms	(“virtual reality” OR VR OR “VR technology” OR “virtual environment*” OR “immersive virtual reality” OR “mixed reality” OR “extended reality” OR “augmented reality”) AND (“music therapy” OR “music therap*” OR music OR MT OR “music intervention*” OR “music-based intervention*” OR “therapeutic music”).
CNKI	Title / abstract / keywords	(“虚拟现实” OR “虚拟现实技术” OR “虚拟环境” OR “沉浸式虚拟现实” OR “混合现实” OR “增强现实” OR “扩展现实”) AND (“音乐治疗” OR “音乐疗愈” OR “音乐干预” OR “音乐聆听” OR “治疗性音乐”)
Wanfang Data	Title / abstract / keywords	(“虚拟现实” OR “虚拟现实技术” OR “虚拟环境” OR “沉浸式虚拟现实” OR “混合现实” OR “增强现实” OR “扩展现实”) AND (“音乐治疗” OR “音乐疗愈” OR “音乐干预” OR “音乐聆听” OR “治疗性音乐”)

No additional document-type filter was applied at the search stage; publication type was assessed during eligibility screening. Although the consumer version of Oculus Rift was released in March 2016, 2015 was retained as the lower boundary of the search period to capture early pilot work, prototype-based applications, and pre-commercial uses of immersive VR in health-related contexts. The initial search was conducted in September 2025 and updated in December 2025 to cover publications available up to 30 November 2025. Database-level preliminary hits from the broad search strings are reported separately in Table 2 and should not be interpreted as the number of records entered into formal screening.

**Table 2:** Preliminary database hits from the broad search strings.

Database	Preliminary database hits from broad search strings
PubMed	135
CINAHL Complete	0
PsycINFO	13
ScienceDirect	218
Web of Science	378
Scopus	204
ProQuest	531
CNKI	143
Wanfang Data	65

**Figure 1:**
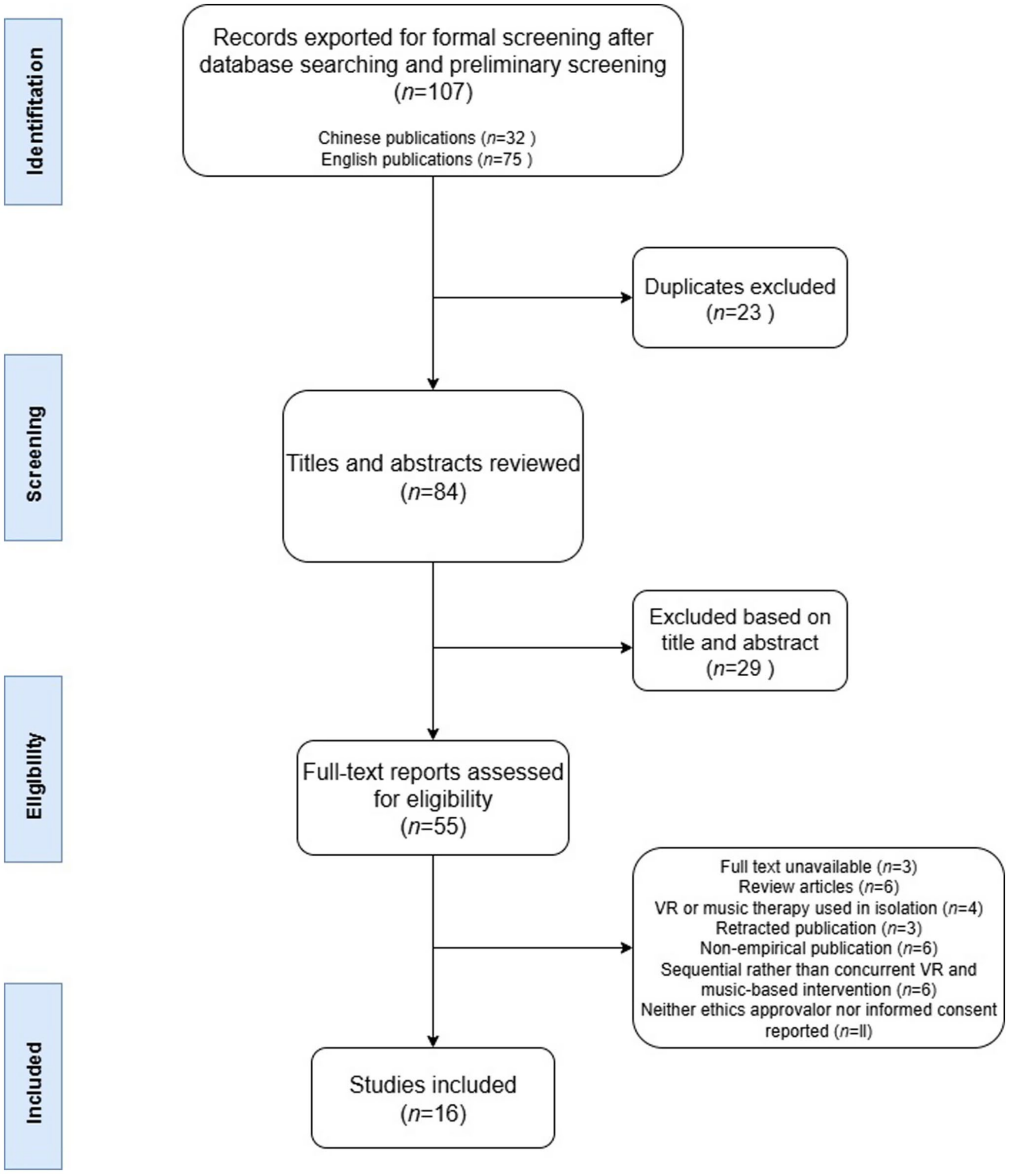
Flow diagram of the search and selection process after applying the revised ethics-reporting eligibility criterion.

Because broad terms such as “music” and “VR” retrieved many clearly irrelevant records when applied to broad field-based searches, the database export process was limited to records that met the predefined publication period and language criteria and showed apparent title/abstract relevance to the concurrent use of VR and music-based intervention. These exported records were then imported into Zotero for duplicate removal and formal screening. To maintain transparency, preliminary broad-search hits are reported separately in Table 2, whereas the 107 records shown in Figure 1 represent the records exported for formal screening after these predefined limits and relevance-oriented record selection procedures. After applying these search limits and record selection procedures, the search yielded 107 records for formal screening, including 75 English-language records and 32 Chinese-language records. Although CINAHL Complete returned no preliminary hits, PsycINFO and ScienceDirect retrieved 13 and 218 preliminary hits, respectively; however, no additional eligible studies from these databases entered the final included set after applying the predefined limits and eligibility criteria.

### 2.4 Eligibility criteria and selection process

Studies were included if they: (1) reported an empirical intervention in which VR was intentionally combined with music therapy or a music-based intervention; (2) described music as an explicit therapeutic or intervention component rather than incidental background sound or unspecified headset audio; (3) targeted a defined population, symptom, therapeutic, rehabilitative, or psychosocial outcome; (4) provided sufficient full-text information for data extraction; (5) reported ethics approval and/or informed consent procedures in the article, thesis, or full-text report; and (6) were published in Chinese or English between 1 January 2015 and 30 November 2025. Both receptive and active music-based approaches were eligible. Administration by a certified music therapist was not required, but therapist involvement, facilitator role, professional credentials, music selection, and intervention mode were extracted when reported.

Studies were excluded if they: (1) used VR or music in isolation; (2) included only incidental audio without a therapeutic music component; (3) were non-empirical publications or duplicate records; (4) were unavailable in full text; (5) did not report interpretable findings; or (6) did not report ethics approval or informed consent procedures. Conference proceedings and theses were retained when they reported empirical data and provided information on ethics approval and/or informed consent, because the purpose of a scoping review is to map an emerging field rather than restrict synthesis to international English-language journal articles only.

The updated search identified 107 potentially relevant records. After duplicate removal, 84 records remained for title and abstract screening. Twenty-nine records were excluded at this stage, and 55 full-text reports were assessed for eligibility. Thirty-nine reports were excluded for the following reasons: unavailable full text (*n* = 3), review articles (*n* = 6), VR or music therapy used in isolation (*n* = 4), retracted publication (*n* = 3), non-empirical publication (*n* = 6), sequential rather than concurrent application of VR and music-based intervention (*n* = 6), and neither ethics approval nor informed consent reported (*n* = 11). The 11 excluded reports are listed in the response to reviewers and were removed from the evidence synthesis. Sixteen studies met the revised inclusion criteria and were included in the descriptive synthesis. The search and selection process is illustrated in Figure 1.

### 2.5 Data collection and analysis

The initial literature search was conducted by one author (LS). All retrieved records were imported into Zotero for reference management and duplicate removal. Two authors (LZ and LS) independently screened titles, abstracts, and full texts against the eligibility criteria, with disagreements resolved through discussion. Data extraction was conducted independently using a standardised extraction form, and extracted data were cross-checked for consistency. The extraction form captured publication type, study design, sample characteristics, country or setting, VR device or platform, intervention duration, music therapy approach, music characteristics, therapist involvement, follow-up, outcome measures, and key findings. No formal critical appraisal or risk-of-bias assessment was undertaken, as this scoping review aimed to map the scope, characteristics, and gaps in the existing literature rather than to evaluate the effectiveness or certainty of evidence. Quality-related and transparency-related reporting issues were noted descriptively when identifiable in the source articles.

### 2.6 Data extraction

In response to the heterogeneity of the included studies, results were synthesised descriptively and organised by study characteristics, population and therapy domain, research design, VR scenario construction, music therapy approach, music selection, therapeutic process, and reported outcomes. During full-text review, studies that did not report either ethics approval or informed consent procedures were excluded. For retained studies, ethics approval and informed consent reporting were checked during eligibility assessment, and information on therapist involvement, facilitator role, professional credentials, music selection, intervention mode, and fidelity-related details was extracted when available and reported descriptively.

For studies with incomplete intervention reporting, extractable information was limited to the available intervention aims, broad intervention format, participant group, and reported outcomes. These studies were retained only as exploratory evidence for mapping purposes and were not used to support strong claims about intervention effectiveness.

## 3 Results

### 3.1 Study characteristics

A total of 16 reports were included in this scoping review. These comprised peer-reviewed journal articles, peer-reviewed conference proceedings, and two master’s theses. Because the aim was to map an emerging interdisciplinary field, non-journal empirical reports were retained when they met the eligibility criteria, provided extractable intervention data, and reported ethics approval and/or informed consent procedures. Reports that did not report ethics approval or informed consent were excluded during full-text eligibility assessment.

Publication activity increased after 2018, although year-to-year differences should be interpreted cautiously because the total number of included studies was small (*n* = 16). The included studies were published across different countries and publication types, with a notable proportion originating from China. Given the small sample size and the search strategy used in this review, geographical patterns are reported descriptively rather than interpreted as evidence of national research attention. Rather than indicating stable annual trends, the distribution suggests that VR-assisted MT remains an early and unevenly developed research area.

Studies were conducted across Europe, Asia, Australia, North America, and South America, with several reports not specifying the study location. China contributed the largest number of studies, followed by the United States. Because the distribution is based on a small and heterogeneous evidence base, geographic patterns are described here only as an indication of where published examples were found, not as evidence of global research dominance or absence. Specific research characteristics are referred to in Table 3.

**Table 3:** Characteristics of the included studies.

Author(s)	Country	Sample characteristics	Device/ system	Aim	Study design	Intervention (duration)	Follow-up	Data collection	Outcomes
Heyse et al. (2022)	Belgium	*n* = 8; (Age >12; >6 months post-acquired brain injury)	Oculus Quest 2	To assess the impact of combined music neglect therapy and VR on Unilateral Spatial Neglect (USN).	Quantitative	Participants performed active music-making tasks, such as playing a virtual xylophone, in a VR environment, using visual and auditory feedback to encourage exploration and movement towards the neglected side of space. The intervention lasted 30 min, three times per week for 2 weeks.	None	Interview; Catherine Bergego Scale; Test for Attention Performance	The multi-sensory VR tool based on Musical Neglect Therapy is a feasible and enjoyable intervention that appeared to encourage patients to actively explore their neglected side, showing promise as a valuable supplement to traditional rehabilitation methods.
Adjorlu et al. (2019)	Denmark	*n* = 4; (Age 18–20; ASD and social anxiety)	Oculus Rift	To explore VR with musical elements for coping with social anxiety in adolescents with ASD.	Qualitative	VR app simulating a 3D music hall with a virtual audience. Participants sing and receive real-time positive feedback (audience of standing/ clapping). Songs/ lyrics were customisable.	None	Observation; Liebowitz Social Anxiety Scale; Immersive Tendency Questionnaire; Presence Questionnaire	VR MT may provide an immersive, engaging experience for adolescents with ASD and social anxiety.
Byrns et al. (2020)	Not reported	*n* = 19; Mean age 72.26; Subjective cognitive decline	FOVE VR Headset	To investigate VR MT effects on positive emotions and cognition in Alzheimer’s patients.	Quantitative	Virtual instruments and fireworks were synchronised with music. EEG tracks emotional response to identify best songs for repetition. Eight 30-s classical excerpts were used.	None	Emotive Epoch EEG headset to track emotions; Positive and Negative Affect Schedule scale; Self-assessment of emotions; questionnaire on cyber-sickness.	The personalised and adaptive system, integrating Virtual Reality, Music Therapy, and EEG feedback, was reported to support mood and cognitive function as a non-pharmacological intervention, although the evidence should be interpreted cautiously.
Brungardt et al. (2021)	United States	*n* = 17; (Age 20–79; Inpatients Cancer, Heart Failure, ESRD)	Oculus Go	To assess feasibility, usability, and acceptability of VR MT for symptom distress in palliative care.	Mixed Methods	Patients co-created music with a therapist, then listened while viewing 360° nature scenes in VR. 20 min/day for 2 days.	None	Edmonton Symptom Assessment System; McGill Quality of Life; System Usability Scale; Interview	VR MT was reported to be feasible, usable, and acceptable, with potential reductions in symptom distress and improvements in QoL.
Dönmez et al. (2025)	Turkey	*n* = 140; (Age 18–65; Tension-type headache)	Oculus Quest 2	To investigate the analgesic and acute mood effects of VR combined with classical music.	Quantitative	EG: Participants viewed a VR forest scene while listening to classical music. CG: Participants received medication and rested in a quiet, dark room. The intervention lasted 7–8 min.	None	Visual Analog Scale; 5-choice ordinal rating scale	VR paired with classical music was reported to reduce pain and improve mood, serving as a potential adjunct to medication-based management.
Impellizzeri et al. (2024)	Italy	*n* = 40; (Age 40–80; Parkinson’s Disease)	CAREN System	To test Neuro-MT + VR (CAREN) for cognitive and executive function improvement.	Quantitative	EG: Treadmill training accompanied by MT and VR percussion activities. CG: Standard functional training. 45 min/session, 3 times/week for 8 weeks.	None	Montreal Cognitive Assessment; Hamilton Rating Scale for Depression; Frontal Assessment Battery	Immersive VR Neuro-MT was associated with improvements in cognitive and executive functions.
Tamplin et al. (2020)	Australia	*n* = 12; Spinal cord injury inpatients	Oculus Rift / HTC Vive / Gear VR	To evaluate a low-latency VR group singing programme.	Quantitative	Phase 1: Online group singing using the vTime VR platform. Phase 2: Comparison of face-to-face singing and VR singing with a therapist.	None	Semi-structured interview; Quebec User Evaluation of Satisfaction with assistive Technology; System Usability Scale; Psychosocial Impact of Assistive devices Scale	High satisfaction. Slight positive psychosocial impact. Potential for distraction from pain/injury.
Kim et al. (2025)	South Korea	*n* = 33; (Age 22–33)	Meta Quest Pro	To compare the efficacy of Gamified Active VR MT with Standard Active and Receptive VR modes	Quantitative	Group 1 (Active VR MT): Drumming interactions with a Non-Player Character (NPC). Group 2 (Receptive VR MT): Passive music listening within the VR environment. Group 3 (Gamified VR MT): Engaging in a rhythm-based drumming game. Lasting for 9 min.	None	Interview; Stress measurements	All modes were reported to reduce stress. User experience varies by type.
Iturri et al. (2023)	Not reported	*n* = 60; Age >18; Palliative care (advanced cancer)	Not reported	To determine if Receptive MT combined with VR reduces symptoms and improves well-being.	Quantitative	EG: Viewing VR nature scenes while listening to preferred music. CG: No intervention. Lasting 30 min.	One Follow-up conducted 48 h post-intervention	Visual Analog Scale; Hospital Anxiety and Depression Scale; Edmonton Symptom Assessment System; Heart rate	The protocol planned to evaluate changes in pain, anxiety, depression, fatigue, sleep disturbance, and well-being after a single-session receptive MT and VR intervention.
Brungardt et al. (2024)	United States	*n* = 17; (Age 20–74; Palliative care inpatients)	Oculus Go	To explore QoL improvement and patient/staff perceptions of VR-MT.	Mixed Methods	Two-day intervention. Patient-therapist co-created music was integrated with 360° VR immersion.	None	Edmonton Symptom Assessment System, revised version; McGill Quality of Life, revised version; interview.	The intervention was feasible, usable, and acceptable for palliative care inpatients.
Huang (2018)	China	*n* = 65; (Age not stated; Ophthalmology patients)	HTC VIVE	To evaluate the efficacy of VR combined with calming music on preoperative anxiety	Quantitative	Patients were equipped with VR headsets to watch the preoperative video, lasting 1 min, 53 s.	1 time	State–Trait Anxiety Inventory; VR Experience Questionnaire	Anxiety reduction appeared more pronounced in younger, unmarried men with lower education and less pronounced in older, married women with higher education.
Fang (2021)	China	*n* = 60; Age not reported; Chronic insomnia	Pico	To test Five Elements (五行) music therapy for insomnia.	Quantitative	EG: Routine care integrated with Traditional Chinese Medicine Five Elements (五行) MT and VR (featuring five specific scenes). CG: Routine care supplemented with MT alone. Weekly sessions for 4 weeks.	Planned but not reported	Pittsburgh Sleep Quality Index; Hamilton Depression Scale.	The combined intervention of VR and Traditional Chinese Medicine Five Elements music therapy was reported to improve sleep quality and sleep duration compared with the control group. It was also associated with reduced anxiety and depression symptoms, with no adverse events reported.
Zhang and Chen (2020)	China	*n* = 86; Age 21–64; Burn patients	HTC Vive Focus	To evaluate the efficacy of VR combined with music for pain relief during wound dressing changes.	Quantitative	Intervention applied 5 min prior to and throughout the dressing change procedure. EG: Viewing VR scenes (Ice or Ocean themes) accompanied by soft music. CG: Routine care. Conducted over three sessions.	None	Numeric rating scale; systolic blood pressure; diastolic blood pressure; heart rate	The combined intervention of VR and music therapy was associated with lower reported pain intensity and more stable physiological indicators than routine care, as measured by pain ratings, heart rate, and blood pressure.
Yang et al. (2025)	China	*n* = 90; healthy adults (45 female; mean age 22 ± 2 years)	VR system / HMD not specified	To investigate the analgesic effects and neural oscillatory mechanisms of music-synchronised VR (MSVR).	Quantitative / RCT	Participants were randomised to MSVR (rhythm-synchronised visuomotor tasks with music), conventional VR (identical tasks with white noise), or non-immersive 2D control in a single session.	None	Pressure pain thresholds; conditioned pain modulation; cold pain intensity/unpleasantness; 64-channel EEG; immersion/realism ratings	MSVR was associated with increased pain thresholds, enhanced conditioned pain modulation, reduced cold pain intensity, and uniquely enhanced parietal alpha oscillations compared with conventional VR or 2D control.
Galery et al. (2025)	Canada	*n* = 41; geriatric outpatients from geriatric and memory clinics	Meta Quest Pro	To evaluate acceptability and effects of a music-based VR intervention on emotion, wellbeing, and mood in geriatric outpatients.	Quantitative / pilot RCT	Intervention group received a one-on-one in-person VR-based music experience; control group received traditional music listening via headphones. Single-session intervention.	None	Retention, satisfaction, Simulator Sickness Questionnaire; PANAS; Warwick-Edinburgh Mental Wellbeing Scale; Visual Analog Mood Scale	The intervention was highly acceptable, with high retention, satisfaction, and tolerance, and was associated with improved positive emotional state, although wellbeing and mood outcomes showed no significant intergroup differences.
Partesotti et al. (2025)	Brazil / Spain	*n* = 5; healthy participants	Extended digital musical instrument / XR-based BehCreative system	To explore the therapeutic and neuroplasticity-related applications of an extended digital musical instrument integrating music, movement, and immersive/digital interaction.	Quantitative / exploratory experimental study	Participants interacted with the BehCreative extended digital musical instrument through embodied movement and sound-generating tasks across repeated sessions.	None	Behavioural/interaction measures; affective self-assessment; neuroplasticity-related assessment	The extended digital musical instrument showed potential to promote engagement, creative learning, positive affective responses, and neuroplasticity-oriented therapeutic applications, but evidence remained exploratory and based on a small healthy sample.

### 3.2 Populations and therapy domains

Participants and therapeutic domains were heterogeneous across the 16 included studies. Participants ranged from adolescents and adults to older adults. Reported ages spanned from late adolescence or adulthood in studies involving social anxiety, stress, or pain to older adult samples in palliative care, geriatric outpatient, and cognitive-rehabilitation contexts, while Huang (2018) and Fang (2021) did not specify participant age. Sample sizes varied considerably, ranging from 4 participants (Adjorlu et al., 2019) to 140 participants (Dönmez et al., 2025). The studies were organised into four broad application domains according to their primary therapeutic aims: psychological and emotional support, pain and symptom management, motor and neurological rehabilitation, and social communication or neurodevelopmental support. These categories were not intended to be mutually exclusive, as some studies addressed multiple outcomes, but were used as an analytical framework to map the evidence more clearly.

The first domain was psychological and emotional support. Studies in this group used VR-assisted music therapy or music-based interventions to reduce stress, anxiety, emotional distress, or broader mental health difficulties. Representative populations included adolescents with ASD and social anxiety, individuals with chronic insomnia, palliative care inpatients, ophthalmology patients, geriatric outpatients, healthy adults, and individuals with subjective cognitive decline or Alzheimer’s disease-related symptoms (Adjorlu et al., 2019; Brungardt et al., 2021, 2024; Byrns et al., 2020; Fang, 2021; Galery et al., 2025; Huang, 2018; Iturri et al., 2023; Kim et al., 2025).

The second domain was pain and symptom management. These studies applied VR-assisted music-based interventions to support distraction, relaxation, pain relief, or symptom management. Populations included patients with headaches, burns, advanced illness, or palliative care needs, as well as healthy adults in experimental pain research (Brungardt et al., 2024; Dönmez et al., 2025; Iturri et al., 2023; Yang et al., 2025; Zhang and Chen, 2020). Although these studies often overlapped with psychological outcomes such as anxiety and mood, their primary intervention purpose was symptom relief.

The third domain was motor and neurological rehabilitation. Studies in this group focused on functional impairments or neuroplasticity-related mechanisms associated with neurological injury, neurodegenerative disease, cognitive decline, or sensorimotor learning. Representative populations included individuals with acquired brain injury and unilateral spatial neglect, patients with Parkinson’s disease or Alzheimer’s disease-related cognitive symptoms, and healthy participants in exploratory neuroplasticity research (Byrns et al., 2020; Heyse et al., 2022; Impellizzeri et al., 2024; Partesotti et al., 2025).

The fourth domain was social communication and neurodevelopmental support. In the retained evidence base, this domain was represented by an exploratory study involving adolescents with ASD and social anxiety, where VR-assisted singing was used to support social exposure and engagement (Adjorlu et al., 2019). A summary of the population groups, therapeutic domains, and intervention purposes is provided in Table 4.

**Table 4:** Summary of population groups, therapeutic domains, and intervention purposes.

Therapeutic domain	Representative populations	Main intervention purposes	Evidence considerations
Psychological and emotional support	Adolescents with ASD and social anxiety; individuals with chronic insomnia; ophthalmology patients; palliative care inpatients; geriatric outpatients; healthy adults; and individuals with subjective cognitive decline or Alzheimer’s disease-related symptoms	Stress reduction, anxiety relief, emotional regulation, relaxation, emotional expression, cognitive-emotional support, and mental health support	Populations and outcome measures were diverse; most findings should be interpreted as exploratory rather than directly comparable.
Pain and symptom management	Patients with headaches, burns, advanced illness, or palliative care needs, and healthy adults in experimental pain research	Distraction, relaxation, pain relief, symptom management, comfort enhancement, and mood improvement	Several studies focused on short-term symptom relief, with limited follow-up evidence.
Motor and neurological rehabilitation	Individuals with acquired brain injury and unilateral spatial neglect, patients with Parkinson’s disease or Alzheimer’s disease-related cognitive symptoms, and healthy participants in exploratory neuroplasticity research	spatial neglect training, gait or movement support, cognitive and executive function training, attention, embodied musical interaction, and neuroplasticity-oriented exploration	Small samples, varied VR tasks, and heterogeneous rehabilitation protocols limited generalisability.
Social communication and neurodevelopmental support	Adolescents with ASD and social anxiety	Social exposure, engagement, communication support, performance-based singing, and feasibility testing	Evidence was limited to one retained exploratory study and should be interpreted cautiously.

### 3.3 Methodological design and intervention characteristics

#### 3.3.1 Methodological design

Regarding methodological design, 13 studies used quantitative methods, including randomised or quasi-randomised controlled designs, pre-post designs, non-randomised comparative designs, and exploratory experimental studies. These studies mainly relied on questionnaire-based outcome assessment, physiological or behavioural measures, or neurophysiological recording, although the specific measures varied substantially because of differences in populations, intervention aims, and outcome domains. One study adopted a qualitative design, using observation and experiential feedback to explore participant experience and feasibility (Adjorlu et al., 2019). Two studies used mixed methods, combining quantitative outcomes with qualitative feedback (Brungardt et al., 2021, 2024). Given this heterogeneity, measurement tools were described rather than directly compared.

#### 3.3.2 Intervention characteristics

Intervention characteristics varied considerably across the included studies. Session duration ranged from less than 2 min (Huang, 2018) to 45 min (Impellizzeri et al., 2024), and the longest reported intervention period lasted approximately 8 weeks (Impellizzeri et al., 2024). However, several studies did not fully report session duration or total intervention exposure. The VR and immersive technologies used included commercial head-mounted displays, rehabilitation systems, mixed-reality devices, extended digital musical instruments, and custom-built virtual environments. Commonly reported devices included Oculus Rift, Oculus Go, Oculus Quest 2, Pico, HTC Vive, FOVE, CAREN, Meta Quest Pro, and XR-based musical systems, while some studies did not specify the equipment used. Software and platforms also varied, including Unity 3D, JackTrip, Second Life®, BehCreative, and bespoke simulation systems. These intervention details are summarised in Table 5.

**Table 5:** Summary of methodological design, intervention characteristics, and reporting considerations.

Dimension	Summary of findings	Reporting considerations
Study design	Quantitative designs predominated, including RCTs, pre-post studies, non-randomised control group designs, and exploratory experimental designs. Fewer studies used qualitative or mixed-methods designs.	The evidence base is mainly outcome-oriented, with limited qualitative process evaluation.
Outcome assessment	Quantitative studies mainly used questionnaires, physiological, behavioural, or neurophysiological measures, but measures varied across populations and therapeutic aims.	Heterogeneous outcome measures prevented direct comparison across studies.
Intervention exposure	Session duration and total intervention length varied widely, from brief single-session exposure to approximately eight-week programmes.	Several studies incompletely reported session duration, frequency, or total intervention dose.
VR technology	Studies used commercial headsets, rehabilitation systems, mixed-reality devices, XR-based extended digital musical instruments, and custom virtual environments.	Hardware and software heterogeneity limits comparability and replication.
Software/platforms	Unity 3D was commonly reported; other platforms included JackTrip, Second Life®, BehCreative, and bespoke simulation systems.	Some studies did not specify software or platform details.
Music-related reporting	Studies varied in how they described music selection, receptive or active mode, therapist/facilitator role, and intervention rationale.	More detailed reporting is needed to clarify the therapeutic contribution of music.

This table summarises information extracted from the 16 included studies listed in Table 3.

### 3.4 Application of VR technology in MT

#### 3.4.1 VR scenario construction

The VR scenarios were synthesised into four broad categories according to their primary therapeutic or functional purpose. First, relaxation-oriented environments used natural or calming scenes, such as forests, oceans, grasslands, auroras, bamboo groves, water towns, or virtual concert experiences, to support emotional regulation, relaxation, and stress reduction. Second, rehabilitation-focused environments used musical instruments, visual cues, haptic feedback, rhythm-synchronised visuomotor tasks, or treadmill-linked scenes to guide motor, cognitive, analgesic, or spatial rehabilitation. Third, social or performance-oriented environments, including virtual classrooms, concert halls, collaborative multi-user spaces, and extended digital musical instruments, were designed to support social communication, performance practice, music-making, therapist-participant interaction, or embodied creative engagement. Fourth, clinical distraction environments, such as simulated operating or wound-care contexts, aimed to redirect attention away from pain, anxiety, or procedural distress.

#### 3.4.2 MT approaches

Among the included literature, nine studies employed active MT or active music-based approaches, or incorporated active music-making and interactive music components within a broader intervention. This methodology emphasises therapeutic change through direct participant engagement in musical actions, such as singing, instrument playing, rhythm-synchronised visuomotor interaction, embodied sound-generating movement, or co-creation of music for use in VR. The specific formats varied widely, including therapist-guided singing or group singing (Adjorlu et al., 2019; Tamplin et al., 2020), active music-making in a VR rehabilitation environment (Heyse et al., 2022), rhythm-synchronised visuomotor tasks (Yang et al., 2025), treadmill-based gait exercises with neurological music therapy components (Impellizzeri et al., 2024), embodied interaction with an extended digital musical instrument (Partesotti et al., 2025), gamified rhythm-based interaction (Kim et al., 2025), and patient-therapist co-creation of music or soundtracks for palliative care VR sessions (Brungardt et al., 2021, 2024).

Receptive MT or receptive music-based approaches primarily involved listening to music within immersive VR environments, often to support relaxation, distraction, emotional regulation, or symptom relief. Several included studies primarily employed this approach, including studies involving classical music, calming music, patient-preferred music, music-based VR experiences, or music paired with clinical distraction scenes (Byrns et al., 2020; Dönmez et al., 2025; Fang, 2021; Galery et al., 2025; Huang, 2018; Iturri et al., 2023; Zhang and Chen, 2020). In most of these studies, participants wore VR headsets in a quiet or clinical environment, viewing visual content while listening to music selected based on personal preference, therapeutic rationale, or a preselected immersive music programme.

Some studies further emphasised therapist presence, dynamic intervention, or adaptive musical interaction. For example, Brungardt et al. (2021, 2024) involved music therapists in creating personalised soundtracks with patients, Tamplin et al. (2020) used therapist-guided group singing, and Impellizzeri et al. (2024) integrated neurologic music therapy techniques with CAREN-based VR rehabilitation. Yang et al. (2025) further highlighted rhythm-synchronised audiomotor integration as a mechanism of analgesic response, while Partesotti et al. (2025) positioned embodied interaction with an extended digital musical instrument as a possible route for creative engagement and neuroplasticity-oriented therapeutic application.

#### 3.4.3 Music selection

Only a small number of retained studies described the musical repertoire in detail. Tamplin et al. (2020) incorporated popular classics, including Bob Dylan’s “Knocking on Heaven’s Door,” Bob Marley’s “Buffalo Soldier,” The Beatles’ “Let It Be,” and Carlos Gardel’s “Por Una Cabeza.” Other studies described music more generally by genre, function, or acoustic quality rather than by specific titles.

Among the retained studies, descriptions of the music were commonly characterised by timbre, genre, function, or intensity. Examples included classical music (Dönmez et al., 2025), calming music (Huang, 2018), traditional Chinese Five Elements music (Fang, 2021), music-based VR concert or listening experiences (Galery et al., 2025), and natural sounds or patient-created soundtracks in palliative care VR-MT (Brungardt et al., 2021, 2024). In contrast, two studies provided more detailed technical specifications regarding rhythm, structure, and intensity. Kim et al. (2025) defined relaxing music through specific parameters: a tempo of 60–90 beats per minute (BPM), soft volume, extended phrasing, and consistent dynamics. This repertoire was further characterised by a predictable structure, an absence of abrupt changes or strong rhythmic accents, and a purely instrumental arrangement. Similarly, Zhang and Chen (2020) focused on acoustic intensity, recommending gentle music presented at a volume of approximately 45 dB.

Some studies indicated that music selection was based on patient or participant preferences, including Iturri et al. (2023) and some palliative care interventions involving collaborative soundtrack creation (Brungardt et al., 2021, 2024). Regarding live or active instrumentation, Impellizzeri et al. (2024) utilised acoustic guitar, sand blocks, and tambourines within a neurologic music therapy framework. Overall, the retained studies varied substantially in the specificity with which music selection, musical parameters, and therapist/facilitator roles were reported.

### 3.5 Therapeutic process and research findings

#### 3.5.1 Motor rehabilitation

One retained study directly addressed neurological motor rehabilitation through VR-assisted musical interaction. Heyse et al. (2022) evaluated tasks using an adjustable virtual xylophone game designed to quantify neglect severity and encourage exploration of the neglected side in individuals with acquired brain injury and unilateral spatial neglect. The study integrated clinical scales, memory tasks, and gameplay elements requiring participants to adapt to environmental triggers characterised by adjustments in the quantity and positioning of keys.

The game design incorporated multiple gamification mechanisms, including falling ball interactions, real-time error correction, and positive feedback; additionally, each task used graduated difficulty levels to ensure appropriate challenge. The results suggested that the tasks engaged participants, evidenced by increased head-turning amplitude during advanced tasks and elevated levels of reported enjoyment throughout the rehabilitation process.

#### 3.5.2 Psychological, pain, and symptom management outcomes

Among the retained studies, Kim et al. (2025) examined VR-assisted music-based interventions in a non-clinical adult sample, comparing receptive VR, active VR, and gamified active music-based approaches rooted in Self-Determination Theory. The study focused on emotional regulation, stress reduction, user engagement, and long-term usage intention.

Kim et al. (2025) reported that all three VR music-based modes reduced daily stress, while the gamified design appeared to enhance user engagement and intention for continued use. These findings suggest that gamification may support adherence when carefully designed, although game elements should be balanced to avoid increasing stress or cognitive load.

Additional retained studies examined emotional or symptom-related outcomes in clinical or quasi-clinical contexts. Huang (2018) evaluated calming audiovisual VR with music for preoperative anxiety, Fang (2021) examined VR combined with Traditional Chinese Medicine Five Elements music for chronic insomnia, and Byrns et al. (2020) explored a personalised VR-music and EEG-feedback approach for emotional and cognitive symptoms associated with Alzheimer’s disease or subjective cognitive decline. Together, these studies suggest potential psychological benefits, but the heterogeneity of populations and outcome measures limits direct comparison.

Several retained studies addressed pain, mood, or symptom-related outcomes in palliative care, burn care, headache management, and experimental pain contexts. Brungardt et al. (2024) reported that VR MT moderately improved symptom burden among adult inpatients receiving palliative care for cancer, heart failure, or end-stage renal disease. Additionally, healthcare team members perceived the intervention as facilitating deeper communication between medical staff and patients.

Zhang and Chen (2020) developed virtual environments, specifically “Snow World” and “Ocean World,” tailored to patient preferences. These environments were complemented by a pre-set music library playing gentle melodies synchronised with each scene’s ambiance, creating a cohesive immersive experience. The study reported that patients receiving this VR-music intervention had lower pain scores during dressing changes, as well as reduced physiological stress markers (heart rate and blood pressure) compared to the standard care group. In the context of headache management, Dönmez et al. (2025) found that supplementing pharmacological treatment with VR and classical music produced a more pronounced reduction in pain intensity across all time points (30, 60, and 120 min). Furthermore, 2 hours after the intervention, the proportion of patients reporting positive emotions rose to 81.4%, markedly exceeding the 31.4% observed in the medication-only control group. Collectively, these findings suggest that VR-assisted music-based interventions may be feasible and may have short-term potential for pain relief and mood improvement. Notably, Iturri et al. (2023) described a study protocol for evaluating a single session of receptive MT combined with VR among hospitalised patients with advanced cancer, with planned outcomes including pain, anxiety, fatigue, sleep disturbance, and psychological well-being. Yang et al. (2025) examined a music-synchronised VR intervention for pain modulation in 90 healthy adults. Compared with conventional VR using white noise and a non-immersive 2D control, the MSVR condition produced stronger increases in pressure pain thresholds, improved conditioned pain modulation, reduced cold pain intensity, and was associated with distinctive parietal alpha oscillatory changes, suggesting a potential mechanism based on rhythm-synchronised audiomotor integration.

While varied in approach, interventions for anxiety consistently utilised immersive environments to induce relaxation; however, their underlying therapeutic mechanisms differed significantly. Adjorlu et al. (2019) adopted an exposure therapy framework targeting social anxiety. By placing participants on a virtual stage to perform singing tasks with controlled audience feedback, the intervention required active engagement, facilitating gradual habituation to the feared social stimulus. In stark contrast, Huang (2018) and Fang (2021) employed distraction and relaxation strategies to manage situational anxiety. Huang (2018) utilised calming audiovisual simulations to divert attention away from preoperative stress, effectively shielding the patient from the anxiety-inducing surgical environment. Similarly, Fang (2021) integrated nature landscapes and music based on the Traditional Chinese Medicine Five Elements (五行) framework to induce physiological relaxation. Thus, whereas Adjorlu’s approach involved confronting the stressor, Huang and Fang focused on buffering against it through sensory immersion.

#### 3.5.3 Cognitive rehabilitation

Byrns et al. (2020) combined VR and MT to address psychological and cognitive symptoms in Alzheimer’s patients. The study reported statistically significant improvements in emotional states and some memory-related outcomes. Specifically, the MT environment reduced negative emotions, such as frustration, while enhancing positive feelings, including relaxation and a sense of self-worth. However, results suggest that while memory performance improved significantly, the intervention’s impact on attention was limited. Conversely, Impellizzeri et al. (2024) integrated neuro-MT into the VR CAREN system to target cognitive and executive functions in Parkinson’s patients. During treadmill walking training, participants engaged with rhythmic auditory stimulation and therapeutic instrumental music combined with perceptual-motor techniques. Supplemented by percussion instruments, metronomes, and customised music, the protocol aimed to facilitate multidimensional improvements in movement fluency, muscle activation, and executive function. The study reported significant improvements in overall cognitive function, executive control, inhibitory control, and visual attention.

#### 3.5.4 User experience and research feasibility

Several retained studies investigated feasibility, acceptability, usability, or user experience of VR-assisted music-based interventions across diverse clinical, geriatric, and exploratory music-technology contexts, including palliative care inpatients, geriatric outpatients, individuals with spinal cord injury, and healthy participants in exploratory XR-based music interaction (Brungardt et al., 2021, 2024; Galery et al., 2025; Partesotti et al., 2025; Tamplin et al., 2020).

Brungardt et al. (2021) employed a mixed-method design to investigate the feasibility, usability, and acceptability of a combined VR and MT intervention for patients diagnosed with cancer, heart failure, or end-stage renal disease. Findings indicate that this therapeutic approach was widely accepted, receiving positive feedback from participants. Concurrently, the study noted that the intervention elicited beneficial physiological changes, including a sense of respite and alleviation of pain, chest tightness, and breathing difficulties. Furthermore, participants offered specific suggestions for improvement, such as enhancing the diversity and interactivity of the VR environment and achieving tighter synchronisation between VR content and musical elements.

Galery et al. (2025) conducted a pilot randomised controlled trial with 41 geriatric outpatients, comparing a one-on-one VR-based music experience with traditional music listening through headphones. The intervention showed high acceptability, retention, satisfaction, and tolerance, and significantly improved positive emotional state, although broader wellbeing and mood outcomes did not show significant intergroup differences.

Tamplin et al. (2020) developed and feasibility-tested an online VR platform for therapeutic group singing among people with spinal cord injury. The study addressed access barriers associated with travel and explored whether low-latency audio and social VR could support real-time group singing from home or clinical settings.

Partesotti et al. (2025) explored an extended digital musical instrument as an immersive music-technology intervention with five healthy participants. The study linked embodied movement, sound generation, and digital/immersive interaction to engagement, creative learning, affective response, and neuroplasticity-oriented therapeutic possibilities, but its small healthy sample means that findings should be interpreted as exploratory.

In summary, the reviewed literature suggests that VR-assisted MT may support diverse therapeutic outcomes, including anxiety and mood regulation, pain and symptom relief, cognitive engagement, motor rehabilitation, neuroplasticity-oriented engagement, and user acceptance. However, these findings should be interpreted as preliminary because included studies remain heterogeneous in population, study design, intervention dose, VR equipment, music selection, therapist involvement, and follow-up reporting.

Follow-up assessment was rarely reported among the included studies; although some protocols planned short-term follow-up, the long-term sustainability of improvements remains unclear in most investigations. Several retained studies explicitly advised caution regarding VR-assisted music-based interventions and called for further validation using larger samples, longer follow-up, or more objective physiological and neurophysiological outcomes.

## 4 Discussion

This scoping review mapped how VR-assisted music therapy has been applied across clinical, rehabilitation, educational, and wellness contexts. Rather than demonstrating definitive effectiveness, the review shows that the field is currently characterised by rapid experimentation, diverse intervention models, and inconsistent reporting. Although the evidence base is promising, it remains preliminary because many studies used small samples, feasibility-oriented designs, short follow-up periods, or incomplete descriptions of music therapy procedures.

The available evidence appears to be concentrated in a limited number of countries and research contexts, suggesting that VR-assisted music therapy remains an emerging field with uneven international development. However, this pattern should be interpreted cautiously because it may be influenced by database coverage, language accessibility, publication practices, and the inclusion criteria used in this review. The heterogeneity of participant groups and therapeutic aims further suggests that VR-assisted music therapy is not yet a standardised intervention model, but rather a flexible set of approaches being adapted across different clinical and psychosocial contexts.

Across the included studies, two broad application directions were particularly evident: psychological and symptom-management support, and neurological or functional rehabilitation. In psychological and symptom-management contexts, VR may enhance presence, engagement, distraction, and access to personalised therapeutic environments, while music therapy may support emotional regulation, expression, relaxation, and interpersonal connection. In rehabilitation contexts, VR may provide task structure, feedback, and embodied interaction, while musical rhythm, timbre, and active music-making may support motor timing, motivation, attention, and cognitive engagement. These mechanisms are theoretically plausible, but they require more direct empirical testing rather than being inferred from outcome changes alone.

A central limitation across the field is that many studies are technology-forward but music-therapy-light in their reporting. VR hardware, software, and scenario design are often described in detail, whereas music selection, tempo, rhythm, intensity, therapist role, intervention fidelity, and theoretical rationale are frequently underreported. This imbalance makes it difficult to determine whether observed effects are attributable to VR immersion, music therapy processes, distraction, novelty, therapist interaction, or their combined action. Future studies should therefore describe both the technological and music-therapeutic components in sufficient detail to support replication and mechanism-based interpretation.

From a practical and clinical perspective, the reviewed evidence suggests that VR-assisted music therapy may have implications for accessibility, personalisation, patient engagement, therapist-patient interaction, and scalable intervention delivery. VR environments may provide immersive therapeutic experiences for patients with limited mobility, restricted access to traditional therapy spaces, or difficulty engaging with conventional face-to-face interventions. The combination of VR and music may also allow visual scenarios, musical materials, interaction modes, and sensory stimulation levels to be adapted to patients’ symptoms, preferences, functional abilities, and therapeutic goals. In addition, VR may extend the ways in which therapists interact with patients by supporting shared virtual environments, guided music-making, co-created soundtracks, and structured multisensory feedback. These features may enhance engagement and support more flexible delivery models in clinical, rehabilitation, educational, or community-based settings.

At the same time, implementation should be approached cautiously. Real-world use depends not only on intervention effects but also on equipment cost, usability, therapist training, infection control, cybersickness management, patient safety, privacy, fidelity of delivery, and scalability. Future research should therefore move beyond feasibility and short-term outcomes by using clearer eligibility definitions, preregistered protocols when appropriate, transparent database-specific search reporting, and more complete descriptions of music therapy components. Studies should also distinguish intentional VR-assisted MT from VR applications that merely include background audio, and should report practical implementation details, including therapist qualifications, session protocols, adverse events, acceptability, barriers to routine use, and longer-term follow-up. Larger controlled studies and mixed-methods process evaluations are needed to determine which patients benefit, which therapeutic mechanisms are active, and whether VR-assisted music therapy can be integrated safely and sustainably into routine healthcare, rehabilitation, educational, or community-based services.

## 5 Strengths and limitations

The main strength of this scoping review is that it brings together a dispersed and interdisciplinary evidence base and maps how VR-assisted music therapy has been defined, implemented, and evaluated across 16 included reports that met the revised eligibility criteria. The review searched both English- and Chinese-language sources and included empirical reports beyond conventional journal articles when sufficient intervention and outcome data were available and ethics approval and/or informed consent procedures were reported. The inclusion of recent studies from pain neuroscience, geriatric mental health, and extended digital musical-instrument research also broadens the map of emerging VR-assisted music-based intervention models.

Several limitations should be considered. First, although the inclusion of Chinese-language publications and non-journal reports broadened the evidence map, it may reduce accessibility for some international readers and complicate independent assessment of methodological and ethical reporting. Second, the revised ethics-reporting criterion led to the exclusion of 11 otherwise relevant empirical reports that did not report ethics approval or informed consent procedures; therefore, the review maps only studies with at least minimal ethical reporting transparency and may not capture the full range of exploratory work in this field. Third, the review may be affected by database coverage and search strategy limitations. Although multiple English- and Chinese-language sources were searched, relevant studies indexed in other databases, published in other languages, or reported outside the searched sources may have been missed. Fourth, no formal critical appraisal or risk-of-bias assessment was conducted. This is consistent with the mapping purpose of a scoping review, but it limits the extent to which conclusions can be drawn regarding intervention effectiveness. Finally, substantial heterogeneity in populations, VR systems, music therapy or music-based intervention approaches, outcome measures, and study designs prevented direct comparison across studies.

## 6 Conclusion

This scoping review suggests that VR-assisted music therapy is an emerging interdisciplinary approach with potential applications in psychological support, pain and symptom management, neurological rehabilitation, cognitive engagement, neuroplasticity-oriented intervention, and social communication. Evidence from the 16 retained studies points to feasibility, acceptability, and possible short-term benefits across several populations and settings. However, the current evidence remains preliminary because of heterogeneous designs, small samples, inconsistent reporting of music therapy components, limited follow-up, and variable methodological transparency. More rigorous and transparently reported research is needed before strong conclusions can be drawn about effectiveness or clinical implementation. Collaboration among music therapists, clinicians, technologists, and patients may help support the development of VR-assisted music therapy as a safe, personalised, and evidence-informed therapeutic approach.

## Data Availability

The original contributions presented in the study are included in the article/supplementary material, further inquiries can be directed to the corresponding author.
